# The Interplay of Morphosyntax and Verbal and Nonverbal Short-Term Memory in Children and Adolescents with Down Syndrome

**DOI:** 10.3390/bs16030315

**Published:** 2026-02-25

**Authors:** Merve Nur Sarıyer Temelli, Selçuk Güven

**Affiliations:** 1 Department of Speech and Language Therapy, Faculty of Health Sciences, Anadolu University, Eskişehir 26470, Türkiye; mnsariyer@anadolu.edu.tr; 2 École d’Orthophonie et d’Audiologie, Université de Montréal, Montréal, QC H2E 2G2, Canada; 3 Centre de Recherche Azrieli du Centre Hospitalier Universitaire Sainte-Justine, Université de Montréal, Montréal, QC H3T 1C5, Canada

**Keywords:** Down syndrome, morphosyntax, short-term memory, Turkish

## Abstract

Down syndrome (DS) is associated with persistent language impairments that extend beyond early childhood, yet evidence from agglutinative languages remains limited. While morphosyntactic weaknesses have been well-documented in Indo-European languages, less is known about how such difficulties are manifested in Turkish, a language in which grammatical relations are primarily marked through morphology. In addition, short-term memory (STM) limitations, particularly in verbal domains, are characteristic of DS and may contribute to language outcomes. This study examined the interaction between morphosyntax and STM in Turkish-speaking children and adolescents with DS. A cross-sectional observational design was employed, including 12 monolingual Turkish-speaking participants with DS (aged 6;7–15;11) and 10 TD peers matched on nonverbal mental age. Participants completed standardized assessments of syntax and morphology, spontaneous language sampling, and STM tasks assessing verbal and visual memory. Children with DS performed significantly below controls on syntactic comprehension and production as well as morphological measures, with larger effects observed for syntax. Noun morphology was less accurate than verb morphology, likely reflecting increased morphophonological complexity. Regression analyses indicated that auditory digit span predicted sentence comprehension, whereas nonword repetition predicted morphological production indexed by mean length of utterance in morphemes. Substantial inter-individual variability was observed within the DS group. These findings suggest that morphosyntactic outcomes in Turkish-speaking children with DS are closely linked to verbal STM capacities and vary considerably across individuals, underscoring the importance of integrated assessment and individualized intervention planning. Future research with larger samples is warranted to confirm and extend these preliminary findings. Findings should be interpreted cautiously due to the limited sample size and are presented as preliminary descriptive evidence. This study provides initial data on Turkish-speaking individuals with Down syndrome.

## 1. Introduction

Down syndrome (DS) is the most common chromosomal disorder in humans, affecting one in every 675 births (Heinke et al., 2021). DS is characterized by a distinctive neurocognitive profile with prominent and persistent language impairments that extend beyond early childhood and affect day-to-day functioning. Difficulties encompass expressive and receptive language, pragmatics, literacy, and broader cognitive and social outcomes (Chapman, 2006; Martin et al., 2009). Although individuals with DS make measurable language gains over time, expressive abilities typically lag behind receptive skills (Abbeduto et al., 2020), and language development is often slower and uneven across domains (Kumin, 2003; Chapman, 2006). Beyond the general association between intellectual disability and communication difficulties, individuals with DS are disproportionately likely to have moderate–severe communication limitations compared to individuals with intellectual disability from other etiologies (Smith et al., 2020). Understanding the mechanisms that shape this language phenotype is essential for designing effective, targeted interventions. Recent neuroimaging studies have further shown that atypical development of frontal and temporal brain regions associated with language and verbal memory contributes to language difficulties observed in individuals with Down syndrome (Dennis & Thompson, 2013). Against this background, understanding the mechanisms that shape this language phenotype is essential for designing effective, targeted interventions and for guiding future research and clinical practice.

### 1.1. Morphosyntax in DS: A Cross-Linguistic Perspective

Morphosyntax refers to the interface between morphology and syntax, encompassing the structural rules governing word formation and sentence organization (Aronoff & Fudeman, 2011). Bound morphology includes grammatical markers such as inflections or derivational suffixes that cannot stand independently and must attach to a lexical base to convey grammatical meaning (Booij, 2012). Turkish is a typologically agglutinative language in which grammatical relations are largely expressed through bound morphemes attached sequentially to word stems (Kornfilt, 1997). A prominent feature of Turkish morphophonology is vowel harmony, a systematic process whereby vowels within a word harmonize according to frontness and roundedness features, contributing to phonological well-formedness and morphological cohesion (Lewis, 2001).

A large body of work shows that morphosyntax—the interface of morphology and syntax—is a core area of difficulty in DS. Across languages, sentence-level syntax (e.g., wh-dependencies, passives, complex clause structures) tends to be more impaired than bound morphology, although bound morphology is not spared (Laws & Bishop, 2003). In production, utterances are often shorter and grammatically simplified, with frequent omissions of main verbs and tense/agreement markers, contributing to reduced language complexity and poorer discourse skills (Eadie et al., 2002; Chapman, 1997; Loveall et al., 2019). Importantly, these patterns are not static; individuals with DS demonstrate growth, but trajectories remain below age expectations without targeted support (Chapman, 2006; Kumin, 2003). Understanding the nature and persistence of these morphosyntactic challenges is critical for designing targeted, evidence-based interventions. Greater awareness of specific areas of difficulty—such as verb morphology, sentence complexity, and verbal working memory limitations—can inform individualized intervention planning, support early identification of language needs, and guide clinicians in selecting appropriate therapeutic strategies to enhance functional communication and academic participation in individuals with Down syndrome.

Complex syntax poses particular challenges for individuals with Down syndrome. Difficulties have been reported not only in passives and interrogatives but also in relative clauses and other embedded constructions. These structures are consistently identified as areas of weakness in the literature (Laws & Bishop, 2003; Thordardottir, 2021; Bol & Kuiken, 1990). At the same time, findings are not uniform across all morphological subsystems and languages. For example, tense morphology in English-speaking children often remains a persistent weakness (Eadie et al., 2002), yet some German-speaking adults with DS have shown morphological performance comparable to mental-age-matched peers, suggesting cross-linguistic variation and task dependencies (Clahsen & Felser, 2006; Chapman et al., 2002).

Evidence across languages also points to differential vulnerability of noun vs. verb morphology in individuals with Down syndrome. For noun inflection, studies report reduced accuracy in grammatical number (dual/plural vs. singular) in Jordanian Arabic (Mashaqba et al., 2020) and difficulties with noun plural formation in German (M. Penke & Witecy, 2021; H. T. Penke, 2018). For verb morphology, children and adolescents with DS frequently under-mark finite verbal agreement in German (H. T. Penke, 2018) and show weaknesses with tense/aspect morphology in Greek (Stathopoulou & Clahsen, 2010; Christodoulou, 2013). More recently, Arabic data also document DS-specific challenges with subject–verb agreement (Mashaqba et al., 2020). At the lexical level, nouns tend to be acquired earlier and produced more accurately than verbs (Caselli et al., 1998), which may interact with morphological learning at the lemma–morpheme interface.

### 1.2. Why Turkish Matters?

Most DS research is based on Indo-European languages with relatively constrained inflectional paradigms. Turkish provides a theoretically informative testbed due to its agglutinative morphology, in which tense, agreement, case, and derivation are encoded by stacked suffixes attached to a stem (Göksel & Kerslake, 2005). The canonical SOV order is flexible, so accurate comprehension relies heavily on morphological markers rather than rigid word order (Kornfilt, 1997). Acquisition studies show that while many inflections emerge early, case and agreement continue to be refined well into the school years (Aksu-Koç & Slobin, 1985; Ketrez, 2012).

Crucially, Turkish inflection is regulated by morphophonological processes: vowel harmony (e.g., ev-e ‘to the house’ vs. köy-e ‘to the village’), consonant alternations (e.g., bebek → bebeğ-i, ACC), and buffer consonants (e.g., araba-y-a, DAT). These processes increase both articulatory and perceptual load, because the selection and surface shape of suffixes depend on phonological context. If phonological processing is weak—a common characteristic in DS—then morphological marking may be indirectly compromised through misperception or misproduction of contrastive segments and allomorphs (Eadie et al., 2002; Kent & Vorperian, 2013; Stoel-Gammon, 2001). Thus, Turkish allows us to test whether the well-documented syntax > morphology asymmetry in DS holds in a language where morphology carries much of the grammatical information, and whether morphophonological complexity differentially affects noun case (often more variable due to harmony and alternations) versus verb inflections. For example, children with Down syndrome may omit tense/agreement markers, produce simplified subject–verb structures, and show inconsistent marking of case or plural morphology. Difficulties are also observed in the use of embedded clauses, passives, and other complex syntactic constructions, resulting in shorter and less morphologically specified utterances.

Cross-linguistic work further underscores morphology-specific vulnerabilities relevant to Turkish. In Arabic, adolescents and young adults with DS show lower accuracy on dual and plural nouns relative to singulars (Mashaqba et al., 2020). Given the frequency and functional salience of pluralization, such asymmetries suggest that processing cost, morphosyntactic abstraction, or working-memory limitations may constrain the application of number features in morphologically rich systems. These converging findings motivate a typologically grounded approach to DS morphosyntax.

### 1.3. Phonology, Motor Speech Factors, and Their Interface with Morphology

Individuals with DS frequently present phonological and oromotor challenges arising from factors such as hypotonia (abnormally low muscle tone) and structural/functional differences in articulators (tongue, palate), with documented articulation deficits and reduced intelligibility (Abbeduto et al., 2007; Barnes et al., 2006; Kent & Vorperian, 2013; Stoel-Gammon, 2001; Cleland et al., 2010; Roberts et al., 2007 Rupela et al., 2010). The consequences extend beyond intelligibility: single-segment morphemes (e.g., English plural/s/, past/t/) can be misperceived/misarticulated, undermining accurate morphological marking (Stoel-Gammon, 2001). In Turkish, where suffix allomorphy is phonology-governed, morphophonological demands may exacerbate these interactions (Güven & Leonard, 2020, 2023). DS language outcomes thus may arise from a multifactorial interplay among morphosyntactic, phonological, and memory constraints rather than from any single locus.

### 1.4. Relationship Between Short-Term Memory and Morphosyntax

There is broad agreement that verbal STM is a relative weakness in DS, exceeding what would be expected from general intellectual level alone (Næss et al., 2015; Buckley, 2008; Fortunato-Tavares et al., 2015; H. T. Penke, 2018). Individuals with DS often struggle on tasks indexing phonological storage and sequential processing (e.g., nonword repetition, digit span, sentence recall)—capacities tightly linked to language learning and real-time morphosyntactic processing (Archibald & Gathercole, 2006; Chapman et al., 1991). By contrast, visual STM (VSTM) is frequently relatively stronger (Jarrold & Baddeley, 1997; Godfrey & Lee, 2018), though it too can support aspects of sentence comprehension and vocabulary. Longitudinal and adolescent studies show that VSTM contributes uniquely to syntax comprehension alongside auditory–verbal STM (Chapman et al., 2002; Miolo et al., 2005), and in young adults receptive vocabulary correlates with visual and verbal STM, indicating that VSTM–language links persist beyond childhood (Prehn-Kristensen et al., 2017).

Grammar and verbal STM exhibit coupled yet distinct growth patterns over time (Næss et al., 2015), motivating analyses that test task-specific STM predictors of morphosyntactic performance. Parallels between DS and developmental language disorder (DLD)—particularly in verbal STM—further underscore mechanistic overlap while acknowledging etiological differences (Laws & Bishop, 2003; Archibald & Gathercole, 2006; Verhagen & Leseman, 2016). In practical terms, limitations in verbal STM may affect children’s ability to retain and process longer or more complex utterances, leading to difficulties in following multi-step instructions, repeating grammatically complex sentences, or maintaining syntactic accuracy during spontaneous speech. For example, a child with reduced verbal STM capacity may omit grammatical markers or simplify sentence structure when attempting to produce longer utterances, which can in turn affect classroom participation and everyday communication.

### 1.5. Heterogeneity

Recent work emphasizes marked inter-individual variability across cognitive and language domains in DS (Onnivello et al., 2022), including cross-sectional developmental trajectories in adaptive functioning and related abilities (Onnivello et al., 2022). This heterogeneity reflects differences in cognitive level, comorbidities, environmental input, and language-specific structural demands. Characterizing within-group dispersion and individual profiles is therefore critical for precision assessment and intervention. However, relatively few studies have systematically examined variability in morphosyntactic and verbal short-term memory (STM) skills within the Down syndrome population, particularly across different linguistic contexts. Most existing research has focused on group-level deficits rather than individual patterns of strengths and weaknesses, limiting our understanding of how heterogeneous cognitive–linguistic profiles influence assessment outcomes and intervention planning (Næss et al., 2015; Finestack & Abbeduto, 2010; Kover et al., 2014).

### 1.6. The Present Study

The present study aims to provide a preliminary descriptive profile of language- and memory-related measures in Turkish-speaking individuals with Down syndrome. The specific aims were as follows:

1. Test the morphosyntactic profile of DS in Turkish, an agglutinative, morphophonologically regulated language.
2. Contrast noun vs. verb morphology by comparing noun case suffixes (with richer morphophonological conditioning) to verb inflections.
3. Evaluate task-specific STM predictors of morphosyntax by testing verbal STM (e.g., nonword repetition, digit/sentence span) and visual STM as predictors of expressive and receptive outcomes.
4. Characterize heterogeneity by quantifying within-group variability and identifying individual relative strengths and weaknesses across morphosyntax and STM.

By situating DS within the typological and morphophonological constraints of Turkish, this study investigates whether language-general patterns of DS extend to a language where morphology carries greater grammatical weight and clarifies how memory resources support or constrain morphosyntactic performance in an under-studied linguistic context.

Research questions

1. What are the morphosyntactic characteristics of Turkish-speaking children with Down syndrome in an agglutinative language such as Turkish?
2. How do children with Down syndrome perform on noun case morphology and verb inflections in Turkish, and are there observable differences between these domains?
3. What are the relationships between morphosyntactic performance and short-term memory measures (verbal and visual STM) in Turkish-speaking children with Down syndrome?
4. How much within-group variability exists in morphosyntactic and STM performance, and what individual patterns of strengths and weaknesses can be identified?

## 2. Method

### 2.1. Participants

A total of 22 individuals participated in the study. The individuals with DS consisted of 12 monolingual Turkish-speaking children and adolescents, comprising 8 boys and 4 girls aged between 6;7 and 15;11 (M = 12;1, SD = 4.4). These participants were recruited from the (removed for anonymization). Participants with Down syndrome were included if they (a) were native speakers of Turkish, (b) had a productive vocabulary of at least 50 words and the ability to produce two-word utterances as reported by their parents, and (c) were able to participate in structured assessment tasks.

Participants were excluded if they had (a) medical conditions that could interfere with testing procedures, (b) severe intellectual disability (IQ < 40), (c) severe uncorrected hearing impairment, or (d) additional neurological or psychiatric diagnoses not commonly associated with Down syndrome. These criteria were applied to ensure that participants could complete the assessment tasks and that language outcomes could be interpreted reliably. Written informed consent was obtained from the parents or legal guardians of all participants prior to participation, in accordance with institutional ethical guidelines.

To assemble a control group of TD children with similar nonverbal mental ages, we assessed the general cognitive abilities of the individuals with DS using the Turkish adaptation of the Stanford Binet Intelligence Scale (Uğurel-Şermin, 1987). Mean age of TD children’ refers to their chronological age; however, matching with participants with DS was based on nonverbal mental age as assessed by the Stanford–Binet, rather than chronological age. TD controls were group-matched to the DS cohort on nonverbal mental age. The mean nonverbal mental age of individuals with DS was 64.2 months (SD = 12.4), while the mean age of TD children was 67.1 months (SD = 17.84). Their mental ages were comparable, as indicated by Welch’s *t* test (t = 0.43, *p* = 0.67). The TD children, six girls and four boys, were recruited from a local kindergarten. All children in this group demonstrated typical language development, as assessed by the Test of Language Development-Primary-4:Turkish (TODIL; Topbaş & Güven, 2017), and met the inclusion criteria for hearing, nonverbal intelligence, and neurological functioning according to reports from parents and/or teachers. Sociodemographic information about the participants is provided in Table 1.

### 2.2. Procedure

We gathered data through various assessments and activities conducted over multiple sessions (ranging from two to three), depending on family availability and the child’s cooperation. These assessments included confirmation of Trisomy 21 diagnosis through genetic and medical reports, as well as information regarding health status and special education services. Collecting these data allowed us to ensure accurate participant characterization and to control for medical or educational factors that could influence language performance.

In addition, parents rated their child’s speech intelligibility for familiar and unfamiliar listeners on a 0–100% scale. These ratings provided complementary functional information about everyday communication abilities and were used in exploratory analyses examining associations between speech intelligibility and morphological outcomes.

All assessments were conducted individually in quiet rooms within rehabilitation or educational centers familiar to the children, in order to minimize anxiety and distraction. Sessions were administered by trained speech and language therapists experienced in working with children with developmental disabilities. Depending on the child’s attention and cooperation level, assessments were completed across two to three sessions, each lasting approximately 30–45 min. Breaks were provided when necessary to maintain engagement and reduce fatigue. Standardized administration procedures were followed for all tasks to ensure consistency across participants.

All procedures employed in the study were performed in accordance with the Declaration of Helsinki. Ethical approval for this study was granted by the (removed for anonymization). All participants were minors; therefore, informed consent was obtained from their parents or legal guardians prior to participation. In addition, verbal and/or behavioral assent was sought from the individuals with DS to ensure that they were willing to take part in the study procedures, in accordance with ethical guidelines for research involving children with developmental disabilities

### 2.3. Measures

#### 2.3.1. Syntax

Syntactic comprehension and expressive syntax were assessed using the Sentence Comprehension and Sentence Repetition subtests of the Turkish version of the Test of Language Development-4 (TODİL; Topbaş & Güven, 2017). TODİL is a standardized language assessment designed for children aged 4 to 9 years and evaluates multiple components of language, including syntax, morphology, semantics, and phonology. The Sentence Comprehension subtest measures children’s ability to understand syntactic structures, whereas the Sentence Repetition subtest provides information about expressive morphosyntactic abilities and verbal short-term memory demands. The test was standardized on a representative sample of 1252 Turkish-speaking children and demonstrates satisfactory psychometric properties (Topbaş & Güven, 2017).

The sentence repetition task comprises 36 sentences, including simple actives, negation, coordination, and complex structures (e.g., adverbial/subordinate clauses) with an average of 6 words per sentence (SD = 1.5, range 3 to 10). Two practice items preceded testing; items were presented in natural prosody; pronunciation errors were not scored as incorrect. Sentences are deemed incorrect if there are any alterations in words or suffixes, while pronunciation errors are not taken into account.

The sentence comprehension test consisted of 30 sentences, incorporating both simple and complex structures, with an average of 4.5 words per sentence (SD = 1.8, range 2 to 9). Each page featured pictures along with two distractors in random order, and the child was tasked with pointing to the picture corresponding to the given sentence. Two probe items were administered before the actual test. The sentences were presented with regular intonation and stress, and if needed, the item was repeated. An item was considered incorrect if an unrelated picture was chosen or if no response was provided.

#### 2.3.2. Morphology

To assess morphology, we employed two approaches. First, we administered the morphological completion subtest of the TODIL (Topbaş & Güven, 2017), a task designed to elicit 38 items spanning verbal, nominal, and derivational morphemes.

Additionally, we gathered spontaneous language samples and subjected them to a distinct morphological analysis. Individual language samples were collected in one-on-one sessions with children, conducted either at the research center or in their kindergarten classrooms, for both the DS and TD groups. These sessions involved age-appropriate materials such as playhouses, toy farm sets, picture books, and colorful drawings to encourage conversation. To ensure diversity and representativeness, samples were collected in two contexts: free play and storytelling. Verbalizations from the children were recorded, transcribed, and coded by the first author using the Turkish version of the SALT 24 software (Acarlar et al., 2004). The sizes of the language samples were similar for both groups. For those with individuals with DS, the average sample consisted of 56.4 complete and intelligible spontaneous utterances (SD = 10.9, range = 49 to 75). The TD children exhibited a comparable mean (48.7; SD = 5.5) and range (41 to 55) in their samples. To assess reliability, 20% of the transcripts (randomly selected) were independently transcribed and coded for morphological accuracy by the researchers of the study. Inter-rater agreement was high for transcription (mean point-to-point agreement = 96%) and for morphological coding (Cohen’s κ = 0.87), indicating strong reliability. Discrepancies were resolved through discussion, and the final dataset reflects consensus coding.

Various measures were derived from the language samples, including mean length of utterance (MLU) for morphemes and MLU for words, along with a morphological error analysis. MLU was computed in two ways: MLU-m as the total number of morphemes divided by the number of complete intelligible utterances, and MLU-w as the total number of words divided by utterances. For example, Çocuk top-u at-tı (‘The child threw the ball’) = 5 morphemes (MLU-m) and 3 words (MLU-w).

We analyzed obligatory contexts for the following: nouns—case (ACC/DAT/LOC/GEN/ABL), number/possessive, person/number agreement; verbs—TAM (e.g., PST/PROG/FUT) and subject–verb agreement. Specifically, verb TAM morphemes included past -DI/-(d)I, progressive -(I)yor, and future -AcAk, and agreement morphemes included 1st/2nd/3rd person singular/plural (e.g., -(I)m, -sIn, -(I)z, -lAr), with vowel-harmony-governed allomorphs. Correct production was defined when the suffix accurately fulfilled its grammatical function and maintained phonological details in the stem + suffix combination. Errors were categorized as substitution of another overt suffix (ACC → DAT bebek-e for target bebek-i), substitution of the unmarked form (nominative araba for target DAT araba-y-a) or omission of the suffix with the stem in the form required if the suffix was present (okul-∅ for target LOC okul-da). Accuracy scores were calculated separately for noun and verb suffix productions, as well as for overall suffix production. Morpheme-based measures may provide a more linguistically sensitive index for Turkish; however, due to methodological consistency with previous DS studies, MLU in words was retained as a descriptive measure.

#### 2.3.3. Phonological STM

To evaluate phonological or verbal short-term memory (STM), we utilized the Turkish Nonword Repetition Test (TNR) (Topbaş et al., 2014), a standardized task designed to assess phonological processing and verbal STM capacity while minimizing lexical knowledge effects. In this task, participants repeated auditorily presented nonwords. The test included 30 items ranging from one to five syllables, consisting of 15 nonwords that violate Turkish phonotactic constraints, 10 nonwords that obey Turkish phonotactic rules, and 5 morphologically complex nonwords. Performance on this task provides an index of participants’ ability to temporarily store and reproduce unfamiliar phonological sequences.

#### 2.3.4. Verbal and Nonverbal STM

To evaluate STM abilities, we utilized the Visual Aural Digit Span Test–Revised (VADS-R) (Karakaş & Yalın, 1995), a Turkish adaptation of the Visual Aural Digit Span Test by (Karakaş & Yalın, 1995). The VADS–R test assesses digit span from both auditory and visual perspectives. During the test, digits were presented individually through either auditory or visual modalities, and the participant was required to respond orally or in written form. The participant had to provide the response in the appropriate modality one second after the presentation of the last digit for a given item. In the oral response mode, the participant repeated the series aloud, while in the written response mode, the participant wrote the digits on an A4-size paper. Nonwriting participants responded orally in all conditions. Scores on the VADS–R test were based on the number of digits in the longest series that the participant could accurately reproduce.

#### 2.3.5. Statistical Analysis

We employed JASP software 18.1 (JASP Team, 2019) to conduct group-level comparisons and regression analyses. Comparisons at the group level between individuals with DS and the control group were conducted using Welch’s *t* test. Correlations were examined using the Spearman Rho correlation coefficient. Effect sizes for Welch’s *t*-tests and Wilcoxon tests were reported using Hedges’ g, and an alpha level of 0.05 was applied to all comparisons. In addition, to investigate whether certain grammatical domains posed disproportionate challenges for individuals with Down syndrome, suffix accuracy was analyzed by grouping suffixes into four categories: case suffixes, noun agreement markers, tense-aspect-mood markers, and subject–verb agreement markers. To investigate whether certain grammatical domains posed disproportionate challenges for individuals with Down syndrome, suffix accuracy scores were grouped into four categories: case suffixes, noun agreement markers, tense-aspect-mood markers, and subject–verb agreement markers. Accuracy scores were compared using a one-way ANOVA. When normality assumptions were violated, the nonparametric Wilcoxon signed-rank test was used as an alternative. Comparisons at the group level between individuals with DS and the control group were conducted using Welch’s *t* test. Correlations were examined using the Spearman Rho correlation coefficient. Effect sizes for Welch’s *t* test and Wilcoxon test were reported with Hedges’ g. An alpha level of 0.05 was applied for all comparisons. Multiple linear regression analysis was utilized to identify the best STM predictor for language outcomes, employing the backward stepping method, wherein all potential predictors were initially included in the model and systematically eliminated until the model identified the most effective predictors. We report regression coefficients together with their 95% confidence intervals to indicate the precision of estimates. Given the modest sample, we also conducted a post hoc sensitivity analysis, which suggested that medium-to-large effects (f^2^ ≥ 0.65) could be detected with 80% power, while smaller effects may be underpowered. To assess whether each individual with DS performed significantly differently in a task compared to their mental age-matched control group, we utilized Crawford and Howell’s *t*-test (Crawford & Howell, 1998). Crawford and Howell’s *t*-test was used to assess whether individual scores in the DS group significantly differed from those of the TD comparison group, as this method is appropriate for small sample sizes and controls for inflated Type I error in single-case analyses. For group-level comparisons between two conditions, to compare participants’ performance on syntax comprehension versus syntax production tasks, we standardized (z-score standardization) the raw scores for the multiple linear regression analysis. There were very few instances of missing data. Only two participants (ID numbers 11 and 12) were unable to complete the VASD-R (Karakaş & Yalın, 1995). As a result, they were excluded from the analyses related to STM.

Overall, results indicated that children with Down syndrome showed weaker morphosyntactic performance compared to mental-age-matched peers, with considerable variability observed within the DS group. Verbal short-term memory measures were significantly associated with morphosyntactic outcomes, whereas visual STM showed weaker or inconsistent associations. Additional analyses further revealed variability across grammatical domains, highlighting heterogeneous linguistic profiles within the DS group.

## 3. Results

The performance of each participant in tasks that measure morphosyntax and STM skills is presented in Table 2.

Do individuals with DS show deficits in morphology and syntax compared to their MA-matched TD peers?

**Figure 1:**
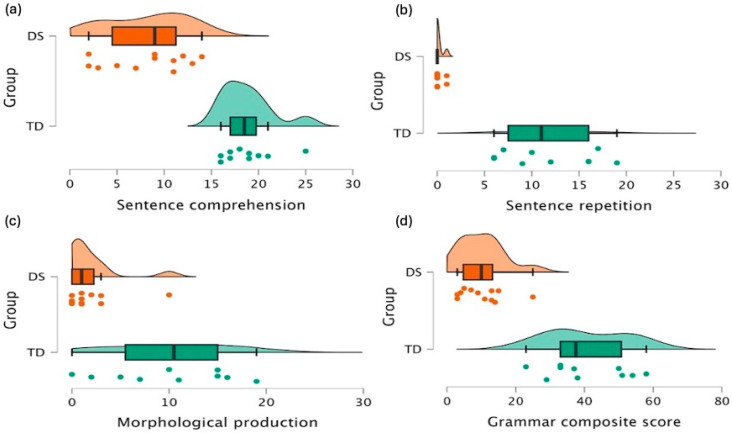
Raincloud Plots Illustrating Raw Scores of the Groups (**a**) Sentence comprehension, (**b**) Sentence repetition, (**c**) Morphological production, and (**d**) Grammar composite score. Orange represents individuals with DS and green represents TD peers. Dots indicate individual participant scores, boxplots represent the median and interquartile range, and the density shapes illustrate score distributions.

To determine whether individuals with DS show deficits in morphology and syntax compared to their MA-matched TD peers, we first compared their sentence comprehension skills (Figure 1a). According to Welch’s *t* test, individuals with DS group performed significantly worse than their MA-matched TD peers (M = 13.03, SD = 4.86), (t(18.9) = 7.02, *p* < 0.001), with a large effect size (g = 0.74). Similarly, the performance of the individuals with DS was significantly worse than the MA-matched TD peers (M = 12.3, SD = 4.87) in the sentence repetition task (Figure 1b) (t(9.1) = 7.47, *p* < 0.001), with a large effect size (g = 0.8). For the Sentence Repetition task, a Mann–Whitney U test confirmed the large group effect (U = 120.0, *p* < 0.001), and Cliff’s δ = 1.0 indicated complete separation between groups (all TD participants outperformed all individuals with DS). These results converge with the parametric analyses and underscore the robustness of the observed floor effects. Furthermore, their morphological performance via the morphological completion task (Figure 1c) showed a similar result: TD group (M = 10.6, SD = 6.5), (t(11.8) = 3.76, *p* = 0.003) with a moderate effect size (g = 0.53). We also wanted to compare the performances as a grammar composite score and converted these three scores to a single grammar composite score, comparing the performances accordingly (Figure 1d). This composite score was calculated to provide a more holistic representation of overall grammatical ability, beyond individual subcomponent analyses. While the individual measures already indicated significant group differences, the composite score confirmed the consistency and robustness of this pattern, offering a concise summary metric that may be particularly useful for future comparisons or clinical applications. Their performances also differed significantly in overall grammar composite score (t(13.3) = 7.3, *p* < 0.001) with a large effect size (g = 0.8).

**Figure 2:**
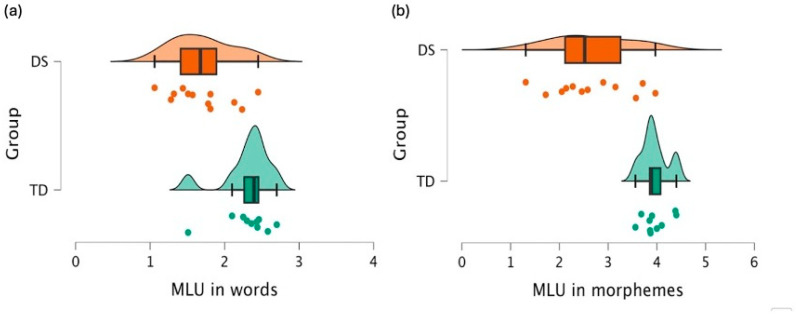
Raincloud Plots Illustrating Morphological Production: (**a**) MLU in Words; (**b**) MLU in Morphemes. Orange represents individuals with DS and green represents TD peers. Dots indicate individual participant scores, boxplots represent the median and interquartile range, and the density shapes illustrate score distributions.

As structured tasks can potentially cause contextual effects or cognitive demands, we additionally assessed participants’ morphosyntactic abilities using a traditional metric for the average length of spoken expressions, MLU, measured in both words (Figure 2a) and morphemes (Figure 2b). This evaluation was conducted through the analysis of spontaneous language samples. According to Welch’s *t* test, individuals in the DS group differed significantly from their MA-matched TD peers in both MLUs in words (M = 2.4, SD = 0.47), (t(19.9) = 3.85, *p* < 0.001), with a moderate effect size (g = 0.53), and in morphemes (M = 4.48, SD = 0.8), (t(13.7) = 5.16, *p* < 0.001), with a moderate effect size (g = 0.6). These findings indicate that both receptive and productive syntax and morphology are significantly reduced than those of their MA-matched peers.

**Figure 3:**
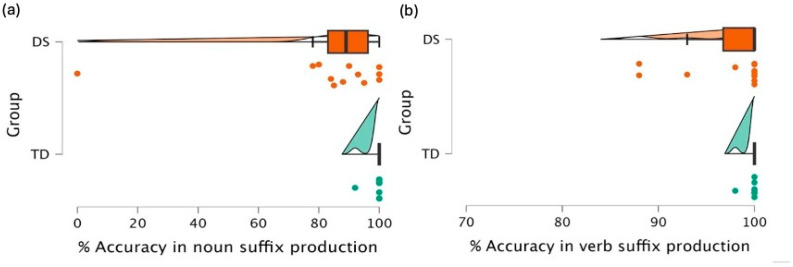
Raincloud Plots Illustrating % Accuracy in (**a**) Noun Suffix Production; (**b**) Verb Suffix Production. Orange represents individuals with DS and green represents TD peers. Dots indicate individual participant scores, boxplots represent the median and interquartile range, and the density shapes illustrate score distributions.

Additionally, certain researchers have suggested that individuals with DS encounter specific challenges with particular suffix categories, such as certain inflectional suffixes in English, such as the 3rd person, past tense -ed, present progressive -ing, and possessives (e.g., Chapman et al., 1998; Katsarou & Andreou, 2022; Vicari et al., 2004 Eadie et al., 2002). To explore this, we first analyzed their speech samples and calculated their accuracy for both noun (Figure 3a) and verb suffixes (Figure 3b).

Welch’s *t* test revealed that individuals with DS produced noun suffixes significantly less accurately than their MA-matched TD peers (*t*(11.1) = 2.15, *p* = 0.05), with a moderate effect size (*g* = 0.45). In addition, parental ratings of speech intelligibility—collected as percentage estimates of how well their child was understood by themselves and by others—showed a moderate positive correlation with noun suffix accuracy (*r* = 0.43, *p* = 0.05). This suggests that children who were rated as more intelligible by their parents also tended to produce noun suffixes more accurately. However, there was no significant difference in their correct use of verb suffixes (t(11.5) = 1.83, *p* = 0.09). To further explore whether individuals with DS faced specific challenges in distinct main suffix groups, we divided accuracy analyses into main suffix groups: case suffixes (M = 95%, SD = 9%), noun agreement (M = 76%, SD = 34%), tense-aspect-mood (M = 95%, SD = 8%), and subject verb agreement (M = 94%, SD = 15%). One-way ANOVA revealed that the differences across these measures were not statistically significant (F(1,44) = 0.33, *p* = 0.567).

Does Morphology Outperform Syntax, or is Comprehension Superior to Production?

Thus far, we have observed that individuals with DS exhibit a distinct pattern when compared to the MA-matched group, both in syntax and morphology. However, we sought to determine whether these individuals display any discrepancy between their performances in syntax and morphology and production vs. comprehension, as suggested by some researchers (Rondal, 1995; Vallar & Papagno, 1993). To address this, we compared their performances in syntactic comprehension, syntactic production, and morphological production using standardized scores. Individuals with DS did not show differences in their performances in sentence comprehension versus sentence repetition (syntactic comprehension versus production) (Wilcoxon z = 0.235, *p* = 0.84) or in syntactic production (sentence repetition versus morphological completion) (Wilcoxon z = 0.392, *p* = 0.72). Nevertheless, we also examined if there are variations in their performances when we compare the accuracies of noun and verb suffixes within the group. The results indicated that they did indeed differ, with their performance in noun suffixes being worse than that in verb suffixes (Wilcoxon z = 2.242, *p* = 0.03, r = 0.3). Since no significant difference was observed between syntactic comprehension and production within the DS group, intervention approaches that strengthen expressive production skills may also indirectly support comprehension through repeated structured practice and increased exposure to grammatical forms. Targeting production in therapy may therefore contribute to broader improvements in functional language use.

Variability in the language and STM Scores within the DS Group

**Figure 4:**
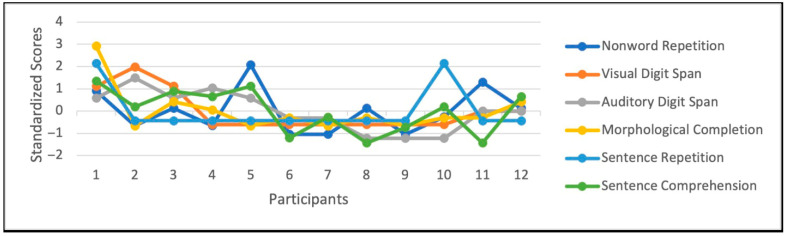
Individual Performance of Participants with DS Across Diverse Language and STM Measures.

While there is a common understanding that individuals with DS are associated with intellectual and speech-language challenges, the extent and nature of these difficulties can vary widely from person to person. We have seen that these individuals indeed show difficulties with morphosyntax as a group; however, as shown in Figure 4, at the individual level, individual performance vary greatly, as reported consistently in many other studies (Chapman et al., 1991; Byrne et al., 1995; Laws & Bishop, 2003).

This inspired us to delve deeper and investigate whether they exhibit consistent patterns or indeed fall behind in both language and STM measures. To accomplish this, we compared their individual-level scores against those of the MA-matched control group using Crawford and Howell’s *t*-test (Crawford & Howell, 1998). The results are presented in Table 3, and all corresponding t and *p* values are provided in Supplementary Table S1.

The findings clearly indicate that at the individual level, syntax challenges, as well as visual and auditory deficits, are evident. Nevertheless, there is variability in individual performance, especially in two morphological measures, especially across the two morphological tasks—morphological completion and language sample analysis—and a noteworthy proportion of individuals with DS appear to possess intact morphological skills.

What best predicts individuals with DS weaknesses in morphosyntax: verbal or nonverbal STM?

Each task used in this study is explicitly linked to the specific linguistic or cognitive domain it is intended to represent. In this context, the nonword repetition task is considered an indicator of phonological STM, while the digit span task reflects verbal working memory capacity. Sentence repetition is interpreted as a measure of syntactic processing, given its reliance on both structural knowledge and memory. Morphological completion and the analysis of language samples are taken as indicators of expressive morphological skills. This alignment allows for a more domain-specific understanding of the observed performance patterns and individual variability across participants.

Another objective of this study was to determine whether verbal or nonverbal STM skills, or both, could account for potential weaknesses in morphosyntax observed in DS individuals. To address this issue, we sought to identify the primary predictors of morphosyntactic weaknesses by comparing the impact of verbal and nonverbal STM. Multiple linear regression was utilized to assess whether phonological STM, visual digit span, and auditory digit span could significantly predict sentence comprehension scores.

By applying the backward stepping method to identify the most influential predictors, the regression model yielded the following equation: Sentence comprehension = 0.141 + 0.448 x (nonword repetition) − 0.535 x (auditory digit span). The overall regression was statistically significant (R^2^ = 0.65, F(2, 7) = 6.47, *p* = 0.02). Remarkably, auditory digit span emerged as a significant predictor (β = 0.55, 95% CI [0.00, 1.10], *p* < 0.05), while nonword repetition showed a trend-level effect (β = 0.47, 95% CI [0.00, 0.94], *p* = 0.08), This result suggested that although phonological STM (verbal STM) was not statistically significant, it still had some effect on sentence comprehension, as did auditory digit span, as evidenced by the significant regression model.

In a separate analysis, we investigated whether verbal and/or nonverbal STM had an effect on expressive syntax, specifically on sentence repetition. Using the backward stepping method, none of the regression models were statistically significant (R^2^ = 0.23, F(3, 6) = 0.62, *p* = 0.6). In other words, neither verbal nor nonverbal STM had an impact on the expressive syntax performance of individuals with DS.

Finally, we explored the influence of verbal and/or nonverbal STM skills on morphological production. This prediction was examined through multiple linear regression. The fitted model, obtained via the backward stepping method, was MLU in morphemes = −0.06 + 0.781 x (nonword repetition). The overall regression was statistically significant (R^2^ = 0.51, F(1, 8) = 8.38, *p* = 0.02), with nonword repetition significantly predicting MLU in morphemes (β = 0.71, 95% CI [0.00, 1.42], *p* < 0.05). These findings offer insights into the distinct contributions of auditory digit span and nonword repetition in predicting morphosyntactic abilities at different linguistic levels. The results indicate that auditory digit span and nonword repetition make distinct contributions to morphosyntactic abilities, likely due to the differing cognitive processes they engage. Notably, nonword repetition was found to significantly predict morphological accuracy in structured tasks, underscoring the importance of phonological STM in the real-time processing and production of grammatical morphemes. Conversely, performance on sentence repetition tasks was more closely linked to auditory digit span, suggesting that this measure reflects not only storage capacity but also the ability to process and integrate syntactic information. Given the variability observed in short-term memory performance, integrating memory training activities may help reinforce language learning and retention. Taken together, these findings suggest that morphosyntactic proficiency is supported by multiple cognitive mechanisms, with specific task demands and linguistic complexity influencing the relative contribution of each component.

## 4. Discussion

The findings of this study should be interpreted in light of the relatively small sample size. Due to the low prevalence of Down syndrome and strict inclusion criteria, the study should be considered exploratory and descriptive rather than confirmatory. Therefore, the results are not intended to provide strong generalizable conclusions but rather to offer preliminary data for Turkish-speaking individuals with Down syndrome, a population for which empirical linguistic evidence remains limited. Developmental interpretations across age should be considered preliminary due to the limited number of participants within each age range.

This study sought to address several research questions. Initially, we explored whether Turkish-speaking individuals with DS encounter challenges in morphosyntax and STM measures compared to peers matched for mental age. Additionally, we aimed to determine whether discrepancies exist between syntax and morphology, as well as between comprehension and production. We also investigated whether individuals with DS exhibit morphosyntactic or STM difficulties at the individual level. Finally, we examined the relationship between language and STM measures.

Taken together, the larger effect sizes on syntactic measures likely reflect the higher integrative load of building hierarchical structure under flexible word order in Turkish, where argument roles must be recovered via morphological cues. In contrast, bound morphology appears relatively less affected at the group level, although individual profiles vary widely. Consistent with this, phonological STM predicted morphological output (MLU-m), whereas auditory digit span related more to sentence comprehension, suggesting partially distinct bottlenecks for morpheme assembly vs. sentence-level integration. This explanatory pattern is consistent with the large composite grammar effect (g = 0.8) and with MLU-based differences derived from spontaneous speech. Considerable inter-individual variability was observed across measures, reflecting the heterogeneous cognitive and linguistic profiles commonly reported in individuals with Down syndrome. Therefore, the findings should be interpreted cautiously and primarily as descriptive patterns rather than uniform group characteristics. These findings should be interpreted with caution, as Turkish is an agglutinative language and word-based MLU may not fully capture morphological complexity. Morpheme-based analyses could provide a more accurate reflection of linguistic productivity in Turkish-speaking individuals with Down syndrome and should be considered in future studies.

One key finding of this study is that, even when compared with the MA matching that was based on nonverbal MA, individuals with DS exhibit significant challenges in both syntax and morphology, spanning receptive and expressive domains. Importantly, these difficulties cannot be solely attributed to cognitive delays. Notably, larger effect sizes associated with both comprehension and production of syntax indicate a more pronounced impact on syntax than on morphology. Complex syntax and the construction of longer sentences emerge as relative weaknesses for children and adolescents with DS, aligning with prior research findings (H. T. Penke, 2018; Laws & Bishop, 2003; Vicari et al., 2004; Lanfranchi et al., 2009; Chapman et al., 1991). Similarly, their performance on both verbal and nonverbal STM measures lagged behind that of previous reports (Conners et al., 2018; Vicari et al., 2004; Martin et al., 2009).

The second objective of this study was to investigate potential differences among language subdomains and between expressive and receptive modalities, as suggested by various researchers (Rondal, 1995; Crawford & Howell, 1998; Abbeduto et al., 2003; Chapman et al., 1998; Snowling, 2008; Fowler et al., 1994; Laws & Bishop, 2003). Initially, we found no distinction between receptive and expressive syntax; both exhibited similar weaknesses, contrary to the prevailing notion that receptive language surpasses expressive language (Deckers et al., 2019; Michael et al., 2012; Abbeduto et al., 2003) but in line with previous research (Lanfranchi et al., 2009). Additionally, we observed no significant difference between syntactic and morphological production skills, which aligns with previous findings indicating that morphology is an area of weakness (H. T. Penke, 2018; Laws & Bishop, 2003; Aksu-Koç & Slobin, 1985). However, notably, compared to their MA-matched counterparts, the effect sizes in syntactic measures were notably larger than those in morphological production tasks. Furthermore, upon examining individual-level performance, we noticed variations in morphology, with a notable proportion of individuals seemingly unaffected in this domain. We attribute this issue to the characteristics of the Turkish language, the native language of the study participants, which is morphologically rich with mostly regular rules. Due to its richness in terms of input and early acquisition of its morphological system (approximately 24 months), Turkish is considered learner friendly. Consequently, it is not surprising to observe that children with neurodevelopmental disorders such as DS benefit from this richness, showing less disparity with their peers compared to other languages as claimed by the theory called “morphological richness account” (Güven & Leonard, 2020). This hypothesis is supported by studies of children with DLD who speak Turkish, demonstrating relatively better performance despite difficulties in morphology (Güven & Leonard, 2020, 2023). Hence, we propose that even though these children encounter cognitive difficulties, they benefit from the complexity and consistency of language. Additionally, their consistent oromotor or speech challenges may impact their performance in this area. Nevertheless, syntax poses a more pronounced difficulty for these individuals.

Another finding from the relevant analyses revealed disparities in the performances of individuals with DS in noun versus verb morphology. Noun morphology exhibited significant differences from that of their MA-matched peers, and within-group analysis suggested significant differences from verb morphology. This finding aligns with numerous studies reporting selective morphological deficits in individuals with DS, as documented by some researchers (Rondal, 1995; Laws & Bishop, 2003; Eadie et al., 2002). To test for selective deficits by grammatical function, we compared case, noun agreement (number/possessive), TAM, and subject–verb agreement. However, our analyses revealed no differences across various major suffix groups with different grammatical functions. The remaining explanation points to the phonological and phonetic influences on the production of suffixes, consistent with findings in other studies (Martin et al., 2009). We also contend that a similar influence extends to the morphosyntactic skills of these individuals. Studies indicate that individuals with DS often experience comorbid speech problems, such as apraxia of speech or phonological problems and mild to moderate hearing problems (Roberts et al., 2007; Jarrold & Baddeley, 2001; Christodoulou, 2013), which impact their language development. It is plausible that the greater difficulties observed in noun suffixes are due to the characteristics of these suffixes. Unlike verb suffixes, noun suffixes in Turkish necessitate various phonological and phonotactic alternations, such as phonetic changes due to vowel and consonant harmony. For example, the word “bebek” (baby) becomes “bebeği” in its accusative form, replacing “k” with “soft g” (a prolongation of the preceding vowel). Similarly, in words ending with a vowel, such as “araba” (car), when the suffix is a single vowel (dative/-a/), a process of syllabification occurs, changing “arabaa” to “arabaya” (to the car). Many similar instances can be found, and such occurrences are observed in numerous other languages characterized by morphological richness. These alternations can add an extra syllable and additional articulatory transitions (e.g., araba + -A → araba-y-a via glide insertion), or trigger lenition (k → ğ) in accusative forms (e.g., bebek → bebeği). Such morphophonological operations increase planning and phonotactic complexity at the word edge, which is plausibly harder under concomitant speech/hearing vulnerabilities. These morphophonological rules pose significant challenges in attaching suffixes to words, especially when accompanied by speech problems or hearing issues. This hindrance may impede the effective learning of these language details. This pattern was observed in children with DLD who speak Turkish, demonstrating specific difficulties related to particular noun suffixes (Güven and Leonard, 2020, 2023). In addition, parental ratings of their children’s speech intelligibility showed a moderate positive association with noun suffix accuracy. Although these ratings are subjective, they provide a useful indicator linking speech clarity with morphological performance. This finding suggests that phonological and phonotactic constraints, as well as potential hearing difficulties, may contribute to morphological accuracy, and underscores the importance of examining these factors more systematically in future research.

Various studies have reported heterogeneity in this population (Snowling, 2008; Byrne et al., 1995; Chapman et al., 1998; Laws & Bishop, 2003). However, this aspect has not received close attention. Therefore, the third objective of this study was to explore the individual performance of children with DS across various morphosyntactic and STM measures, aiming to gain a better understanding of their unique profiles. At the individual level, we observed a nuanced picture distinct from group comparisons. While most of the individuals exhibited significant difficulties with syntax (both comprehension and production) and nonverbal and verbal STM skills compared to their MA-matched peers, their performances varied in morphological measures. These findings suggest two important points. First, morphological performance may be significantly influenced by factors such as accompanying speech problems, and the severity of these challenges varies from one individual to another. Secondly, this study emphasizes the significance of considering individual variabilities when interpreting language and STM results. This approach allows for the identification of individual strengths and weaknesses, which in turn supports the design of developmentally appropriate and functionally relevant interventions. By tailoring goals to each child’s specific morphosyntactic and memory profile, clinicians can improve the effectiveness and efficiency of therapy, ultimately leading to better language outcomes, which also ensures the characterization of precise profiles and the customization of intervention plans accordingly.

The final objective of this study was to examine whether the verbal or nonverbal STM skills of individuals with DS predict their performance on morphology and syntax measures. Our findings revealed that verbal STM skills are significantly impaired, and importantly, they also significantly predict the sentence comprehension and morphological production performance of individuals with DS. However, there was no significant prediction observed with visual STM. These results contribute to existing research, highlighting the deficits in both visual and verbal STM skills among these individuals (Conners et al., 2018; Vicari et al., 2004; Lanfranchi et al., 2009; Carney, 2018). Furthermore, our findings support the idea that, contrary to Chapman et al. (2002), verbal STM, rather than visual STM, is a predictor of sentence comprehension skills (Chapman et al., 1991). However, we also recognize the potential influence of visual memory in the progression of written language and reading skills. In recent discussions (Krishnan et al., 2017), it has been noted that tasks involving oral production, such as verbal short memory tasks like nonword repetition, are influenced by the oromotor abilities of participants. This impact is particularly plausible for this specific group. Hence, it remains uncertain if the difficulties stem from an STM problem unless the experimental conditions are carefully controlled.

In summary, this study contributes to the existing body of literature investigating the language and STM of children and young people/teens (6–15 years) with DS. It explores various aspects, including potential relationships, differences in different language subdomains and modalities, and individual performance variabilities. Our findings indicate that individuals with DS encounter greater challenges in syntactic comprehension and production than in the morphology domain. It is crucial to consider individual differences, as some individuals do not exhibit issues with morphological production. The majority also experience difficulties in both verbal and nonverbal STM skills. Notably, only verbal STM skills predict challenges in both syntactic comprehension and morphological production.

These findings suggest that clinicians or educators working with these individuals should equally prioritize the assessment and intervention of both language and STM skills. Additionally, when interpreting evaluation results, particularly concerning difficulties with morphology, caution should be exercised, and potential influences of speech sound issues or hearing problems should be taken into account. Finally, further research is warranted to replicate these findings with a larger sample size and incorporate both structured and naturalistic measures to address the potential effects of structured tasks, such as increased cognitive demands. Future studies should also explore developmental trajectories across different age groups, examine cross-linguistic patterns in morphologically rich languages, and investigate how intervention approaches targeting both language and memory processes influence long-term communication outcomes in individuals with Down syndrome.

## 5. Limitations of the Study

While this study provides valuable insights into the morphosyntactic and memory profiles of individuals with DS, several limitations must be acknowledged. First, the relatively small sample size (n = 22), particularly within subgroups, limits the generalizability of the findings. Although nonverbal mental age matching was employed to ensure cognitive comparability between groups, the age range within the DS group (6;7–15;11) was wide, introducing potential developmental variation that could have influenced performance. Future research with larger and more age-homogeneous samples is needed to more precisely trace developmental trajectories. The relatively small sample size limits statistical power and external validity. The wide age span combined with a small sample size limits age-based interpretations. Future studies with larger and age-stratified samples are needed to confirm the present findings. The relatively high variability within the DS group further limits the interpretation of group-level patterns and highlights the heterogeneous nature of this population.

Second, the cross-sectional design restricts conclusions about developmental change. Although syntax and morphology were measured using both standardized tools and spontaneous language samples, task demands may have interacted with attentional, behavioral, or motivational factors, particularly in children with DS.

Third, although verbal and nonverbal STM were assessed with established tools, the psychometric properties of some tasks for individuals with intellectual disabilities warrant further validation. A finer-grained analysis of STM subcomponents (e.g., storage vs. processing) and their relation to specific language domains would strengthen future work. Similarly, while regression analyses suggested distinct contributions of auditory digit span and nonword repetition, the wide 95% CIs reflected the modest sample size, and these findings should therefore be considered exploratory and pending replication.

In addition, despite efforts to control for hearing and neurological conditions, other factors—such as socio-economic background, home language environment, and educational input—were not fully accounted for and may have contributed to individual differences. Participants were recruited from rehabilitation and special education centers located in urban areas. Detailed socioeconomic and parental education information was not systematically collected; therefore, these variables could not be controlled for in the analyses and should be considered in future research. Finally, some sections of the manuscript used terminology such as “DS individuals,” which does not align with person-centered language; future work will revise phrasing to reflect more inclusive practices.

In light of these limitations, the findings should be interpreted with caution, and further longitudinal, multi-method studies are warranted to deepen understanding of the complex interplay between language and memory in individuals with DS. Despite its limitations, the study provides preliminary descriptive data that may serve as a useful reference for Turkish clinicians working with individuals with Down syndrome. These findings may contribute to increased awareness of linguistic variability and support clinical assessment and intervention planning.

## 6. Clinical Implications

From a clinical perspective, the dissociation we observed between morphological accuracy and syntactic performance, as well as the distinct contributions of phonological and verbal STM, highlights the need for individualized intervention planning in DS. For children whose difficulties are primarily morphophonological, therapy may benefit from targeted practice with inflectional alternations and syllabic complexity, whereas for those with greater syntactic challenges, intervention may need to emphasize sentence-level comprehension and integration. The identification of within-group variability also underscores the importance of assessment approaches that move beyond group averages to capture individual strengths and weaknesses, thereby supporting more tailored therapeutic goals.

## 7. Conclusions

This study provides preliminary evidence on the relationship between morphosyntax and STM in Turkish-speaking children and adolescents with DS. Compared to nonverbal mental-age-matched peers, participants with DS showed significant weaknesses in both syntactic comprehension and production, with larger impairments in syntax than in morphology. Within morphology, noun suffixes were produced less accurately than verb suffixes, likely reflecting the greater morphophonological complexity of Turkish noun marking.

Verbal STM was closely related to language performance: auditory digit span predicted sentence comprehension, while nonword repetition predicted morphological productivity (MLU in morphemes). Visual STM showed weaker and less consistent associations with morphosyntax. These findings suggest that different components of verbal STM support different levels of grammatical processing.

Considerable inter-individual variability was observed, indicating that language and memory profiles in DS are heterogeneous and should be considered in assessment and intervention planning. Clinically, the results highlight the importance of integrating language and verbal STM support in intervention, while also considering speech and morphophonological factors that may affect morphological accuracy.

Given the small sample and cross-sectional design, these findings are preliminary. Larger, longitudinal studies are needed to confirm and extend these results, particularly in morphologically rich languages like Turkish.

## Figures and Tables

**Table 1:** Characteristics of the participants.

DS Group (*n* = 12)	
Type of DS	n (%)
Trisomy	12 (100)
Chronological age in years	M, SD (range)
	12;1, 4;4 (6;7–15;11)
Biological sex	n (%)
Male	8 (67)
Hearing status	n (%)
Normal hearing ^1^	12 (100)
Nonverbal Mental Age in Months	M, SD (range)
	64.2, 12.4 (48–84)
Intelligibility ^2^	n (%)
Mild intelligibility problem	10 (83)
Moderate intelligibility problem	2 (17)
TD Group (n = 10)	
Chronological age in years	M, SD (range)
	67.1, 17.84 (48–96)
Biological sex	n (%)
Female	6 (60)

^1^ The hearing status of six individuals at the DILKOM Research Center was assessed through a pure-tone hearing test. Parents of the remaining participants confirmed that their children had undergone recent hearing tests and had successfully passed them. ^2^ Intelligibility was assessed by asking parents to indicate whether they believed their children had any issues with clarity of speech. Parents were asked to rate this both categorically and as a percentage. For descriptive purposes, intelligibility levels were classified as mild difficulties when speech was understood approximately 75–90% of the time, moderate difficulties when intelligibility ranged between 50 and 75%, and severe difficulties when less than 50% of speech was understood.

**Table 2:** Descriptive Analysis of Morphosyntax and STM Measures (Raw Scores) in the DS Group.

ID	Sentence Comprehension (n = 30)	Sentence Repetition (n = 36)	Morphological Production			Nonword Repetition (n = 30)	Visual Digit Span (n = 18)	Auditory Digit Span (n = 18)
			Completion Task (n = 38)	MLU in				
				Words	Morphemes			
1	14	1	10	1.81	2.9	5	2	4
2	9	0	0	2.235	3.97	1	3	6
3	12	0	3	2.45	3.57	3	2	4
4	11	0	2	1.81	2.275	1	0	5
5	13	0	0	2.13	3.71	8	0	4
6	3	0	1	1.28	1.72	0	0	2
7	7	0	0	1.32	2.05	0	0	2
8	2	0	1	1.44	2.14	3	0	0
9	5	0	0	1.06	1.31	0	0	0
10	9	1	1	1.78	3.15	2	0	0
11	2	0	1	1.51	2.46	6	-	-
12	11	0	3	1.57	2.58	3	-	-
Mean(SD)	8.2(4.3)	0.2(0.4)	1.8(2.8)	1.7(0.4)	2.7(0.8)	2.7(2.6)	0.7(1.2)	2.7(2.2)

Note. Two data points are missing for both visual STM and verbal STM because the respective participants were unable to complete the tasks.

**Table 3:** Individual-level results in various language and STM measures.

ID	Syntactic Comprehension	Syntactic Production	Morphological Production		Phonological STM	Visual Digit Span	Auditory Digit Span
			Completion Task	Language Sample			
**1**	**√**	**√**	**√**	**√**	**√**	**X**	**X**
**2**	**X**	**X**	**X**	**X**	**X**	**X**	**√**
**3**	**X**	**X**	**√**	**X**	**X**	**X**	**X**
**4**	**X**	**X**	**√**	**√**	**X**	**X**	**X**
**5**	**X**	**X**	**X**	**X**	**√**	**X**	**X**
**6**	**X**	**X**	**√**	**X**	**X**	**X**	**X**
**7**	**X**	**X**	**X**	**X**	**X**	**X**	**X**
**8**	**X**	**X**	**√**	**X**	**X**	**X**	**X**
**9**	**X**	**X**	**X**	**√**	**X**	**X**	**X**
**10**	**X**	**X**	**√**	**X**	**X**	**X**	**X**
**11**	**X**	**X**	**√**	**X**	**√**	**X**	**X**
**12**	**X**	**X**	**√**	**√**	**X**	**X**	**X**

X: Significantly different; √: No difference compared to MA-matched peers. In the table, red X symbols indicate unsuccessful performance, whereas green check marks (√) indicate successful performance in language and short-term memory measures.

## Data Availability

Data is unavailable due to privacy or ethical restrictions, but a statement is still required.

## Supplementary Materials

The following supporting information can be downloaded at: https://www.mdpi.com/article/10.3390/bs16030315/s1, Table S1: Comparison of participants’ single test scores to the control group using Crawford and Howell’s *t*-test.
