# Supplementary material for: The Interplay of Morphosyntax and Verbal and Nonverbal Short-Term Memory in Children and Adolescents with Down Syndrome
Source: Behav Sci (Basel). 2026 Feb 25;16(3):315. doi: 10.3390/bs16030315 (PMC13024546; doi:10.3390/bs16030315)
Supplement: Supplementary file 1 [file behavsci-16-00315-s001.zip › behavsci-4125203-supplementary.pdf]

Supplementary Materials

Table S1. Comparison of participants' single test scores to the control group using Crawford and Howell's t-test.

ID	Sentence Comprehension		Sentence Repetition		Morphological Completion		MLU in Morphemes		MLU in Words		Noun Suffix Production		Nonword Repetition	
	t	***p***	***t***	***p***	***t***	***p***	***t***	***p***	***t***	***p***	***t***	***p***	***t***	***p***
**1**	-1.68	0.07	-2.12	<.05	0.00	0.5	-3.77	<.001	-1.51	0.15	-1.61	0.13	-1.72	0.06
**2**	-3.44	<.001	-2.32	<.05	-1.79	<.05	0.04	0.9	-0.23	0.8	-8.15	<.001	-2.25	<.05
**3**	-2.38	<.05	-2.32	<.05	-1.25	0.12	-1.39	0.19	0.40	0.7	-5.84	<.001	-1.99	<.05
**4**	-2.74	<.01	-2.32	<.05	-1.43	0.09	-6.00	<.001	-1.47	0.17	0.31	0.7	-2.25	<.05
**5**	-2.03	<.05	-2.32	<.05	-1.79	<.05	-0.89	0.39	-0.54	0.6	-4.30	<.001	-1.33	0.1
**6**	-5.54	<.001	-2.32	<.05	-1.61	0.07	-7.97	<.001	-3.03	<.01	-38.12	<.001	-2.38	<.05
**7**	-4.14	<.001	-2.32	<.05	-1.79	<.05	-6.80	<.001	-2.91	<.01	-7.38	<.001	-2.38	<.05
**8**	-5.89	<.001	-2.32	<.05	-1.61	0.07	-6.48	<.001	-2.56	<.05	-2.38	<.05	-1.99	<.05
**9**	-4.84	<.001	-2.32	<.05	-1.79	<.05	-9.43	<.001	-3.67	<.05	0.31	0.7	-2.38	<.05
**10**	-3.44	<.001	-2.12	<.05	-1.61	0.07	-2.88	<.01	-1.56	0.14	-5.46	<.001	-2.12	<.05
**11**	-5.89	<.001	-2.32	<.05	-1.61	0.07	-5.34	<.001	-2.35	<.05	-3.53	<.001	-1.59	0.07
**12**	-2.74	<.01	-2.32	<.05	-1.25	0.12	-4.91	<.001	-2.18	<.05	0.31	0.7	-1.99	<.05
